# Discovery of a Flavonoid FM04 as a Potent Inhibitor to Reverse P-Glycoprotein-Mediated Drug Resistance in Xenografts and Improve Oral Bioavailability of Paclitaxel

**DOI:** 10.3390/ijms232315299

**Published:** 2022-12-04

**Authors:** Jason W. Y. Kan, Clare S. W. Yan, Iris L. K. Wong, Xiaochun Su, Zhen Liu, Tak Hang Chan, Larry M. C. Chow

**Affiliations:** 1 Department of Applied Biology and Chemical Technology and State Key Laboratory of Chemical Biology and Drug Discovery, Hong Kong Polytechnic University, Hong Kong SAR, China; 2 Department of Chemistry, McGill University, Montreal, QC H3A 2K6, Canada

**Keywords:** flavonoids, P-glycoprotein, multidrug resistance, modulator, oral bioavailability

## Abstract

Biotransformation of flavonoid dimer **FD18** resulted in an active metabolite **FM04**. It was more druggable because of its improved physicochemical properties. **FM04** (EC_50_ = 83 nM) was 1.8-fold more potent than **FD18** in reversing P-glycoprotein (P-gp)-mediated paclitaxel (PTX) resistance in vitro. Similar to **FD18**, **FM04** chemosensitized LCC6MDR cells towards multiple anticancer drugs by inhibiting the transport activity of P-gp and restoring intracellular drug levels. It stimulated the P-gp ATPase by 3.3-fold at 100 μM. Different from **FD18**, **FM04** itself was not a transport substrate of P-gp and presumably, it cannot work as a competitive inhibitor. In the human melanoma MDA435/LCC6MDR xenograft, the co-administration of **FM04** (28 mg/kg, I.P.) with PTX (12 mg/kg, I.V.) directly modulated P-gp-mediated PTX resistance and caused a 56% (*, *p* < 0.05) reduction in tumor volume without toxicity or animal death. When **FM04** was administered orally at 45 mg/kg as a dual inhibitor of P-gp/CYP2C8 or 3A4 enzymes in the intestine, it increased the intestinal absorption of PTX from 0.2% to 14% in mice and caused about 57- to 66-fold improvement of AUC as compared to a single oral dose of PTX. Oral co-administration of **FM04** (45 mg/kg) with PTX (40, 60 or 70 mg/kg) suppressed the human melanoma MDA435/LCC6 tumor growth with at least a 73% (***, *p* < 0.001) reduction in tumor volume without serious toxicity. Therefore, **FM04** can be developed into a novel combination chemotherapy to treat cancer by directly targeting the P-gp overexpressed tumors or potentiating the oral bioavailability of P-gp substrate drugs.

## 1. Introduction

The development of multidrug resistance (MDR) has limited the successful outcome of cancer chemotherapy. Overexpression of a family of ATP-binding cassette (ABC) transporter is one of the factors causing clinical MDR. P-glycoprotein (P-gp, MDR1 or ABCB1) is a member of the ATP-binding cassette (ABC) transporter proteins and it can pump a wide variety of structurally different therapeutic agents from the cells and finally result in treatment failure [1,2,3,4]. 

The broad substrate spectrum of P-gp highly restricts the use of potent anticancer drugs in treating cancer. Paclitaxel (Taxol, PTX), doxorubicin (DOX), mitoxantrone, vinblastine and vincristine are P-gp substrates and susceptible to P-gp-mediated efflux in cancer cells. A strategy has been suggested to overcome the P-gp mediated MDR phenomenon in the clinic by the use of the P-gp modulator in combination with anticancer drugs. 

The first P-gp modulator discovered was verapamil, a calcium channel inhibitor for treating high blood pressure. It was found that it could interact with P-gp as a modulator in 1981 [5]. The lack of specificity and the relatively low potency of verapamil towards P-gp did not lead to encouraging results. Further structural modification of verapamil had led to the second-generation modulator dexverapamil [6,7]. Its potency and specificity had been improved. However, it displayed modest activity in clinical trials [8]. The third generation of modulators included elacridar (also known as GF120918) [9,10], XR9576 [11] and LY335979 [12,13]. They were effective at the nanomolar range and demonstrated a tolerable safety profile, but no improvement in anticancer efficacy was noted in the clinical trials [14,15,16]. Therefore, so far, all P-gp modulators failed in the clinical trials. A factor for the lack of success may be that the patient selection was not based on the prospective evaluation of the expression of the drug transporters. An example is a trial that evaluated P-gp inhibitors in non-small cell lung cancer, transporters such as Multidrug Resistance Associated Protein 1 (MRP1) or Breast Cancer Resistance Protein (BCRP), other than P-gp, might have accounted for the drug resistance [3,17]. Other factors contributing to the failure may be the enhanced toxicity at non-target sites due to the inhibitor or drug–drug interaction between the inhibitor and the anticancer drug [18]. Future development of inhibitors of ABC transporters should focus on potency, specificity and safety. 

**Figure 1:**
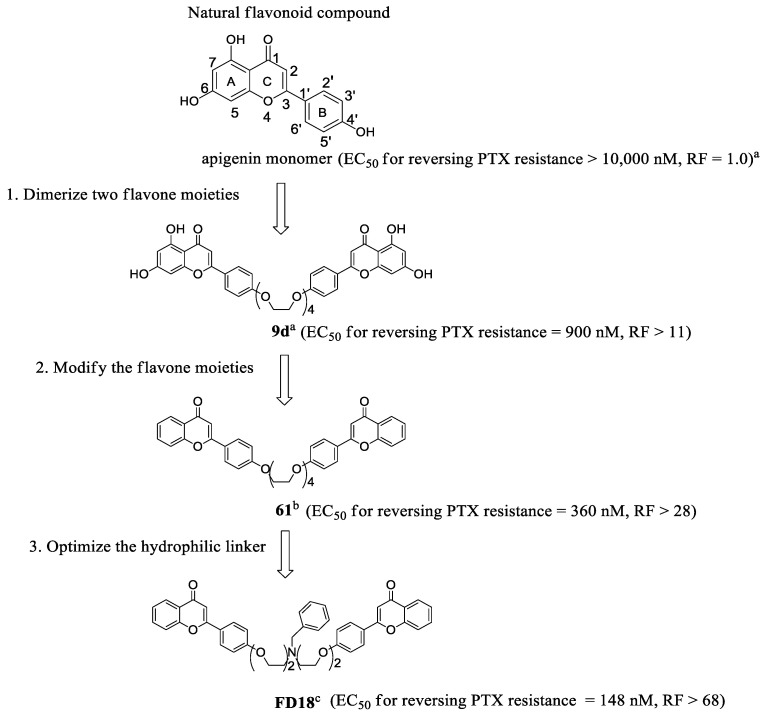
Chemical structures and P-gp modulating activities of different generations of synthetic flavonoids. Three generations of synthetic dimeric flavonoids were designed and synthesized to reverse P-gp-mediated MDR. EC_50_ was the effective concentration of a modulator at which the IC_50_ of an anticancer drug can be reduced by half in the cancer cell line. RF (relative-fold) was the ratio of EC_50_ of apigenin to EC_50_ of different synthetic flavonoids. ^a^ Structure and EC_50_ of apigenin and **9d** had been reported in 2006 [21]. ^b^ Structure and EC_50_ of **61** had been reported in 2009 (Reprinted/adapted with permission from ref. [22], copyright © 2009 by WILEY-VCH Verlag GmbH & Co. KGaA, Weinheim. ^c^ Structure and EC_50_ of **FD18** had been reported in 2012 [23].

Recently, we have turned to flavonoids as the scaffold to develop safe, potent and selective inhibitors of ABC transporters [19]. Apigenin is a safe dietary flavonoid and it has been demonstrated to enhance the DOX uptake at 60 µM and diminish IC_50_ of DOX at 20 µM in P-gp overexpressing leukemia cells (CEM/ADR5000) [20]. In order to further enhance the potency of apigenin, we took advantage of the pseudo-dimeric structure of P-gp by using bivalent interactions [19]. A new class of potent and non-toxic P-gp modulators was formed by conjugating two natural apigenins together with different lengths of polyethylene glycol (PEG) as the linker for reversing P-gp-mediated MDR (Figure 1) [21]. Flavonoid dimer **9d** with 4 PEG units in the linker showed at least an 11-fold improvement as compared to apigenin (Figure 1) [21]. Next, the removal of the OH groups from ring A of the **9d** resulted in a more potent compound **61** which displayed a RF > 28 [22]. Next, we modified **61** by introducing a hydrophilic amine-linked benzyl ring to the linker to obtain compound **FD18** which exhibited the highest potency with EC_50_ of 148 nM and RF > 68 (Figure 1) [23]. Flavonoid dimer **FD18** with adequate potency and acceptable safety was demonstrated to significantly reverse the P-gp-mediated MDR in tumor-bearing mice [24]. 

In this study, an active metabolite of **FD18**, the amine-containing flavonoid monomer **FM04**, was discovered as a potent P-gp modulator. The in vitro assay revealed that **FM04** (EC_50_ = 64 to 83 nM) was more potent than the parent compound **FD18** (EC_50_ = 116 to 148 nM) for P-gp modulation. **FM04** is likely to possess better drug-like properties with a smaller molecular weight (MW of **FM04**: 415 vs. MW of **FD18**: 724) and improvement in aqueous solubility (CLogP of **FM04**: 4.9 vs. CLogP of **FD18**: 9.0). The in vivo efficacy of **FM04** in reversing P-gp-mediated drug resistance and its improvement of oral bioavailability of paclitaxel (PTX) were successfully demonstrated. 

## 2. Results

### 2.1. Metabolism of **FD18** and Metabolites Identification in Rat and Human Liver Microsomes

**Figure 2:**
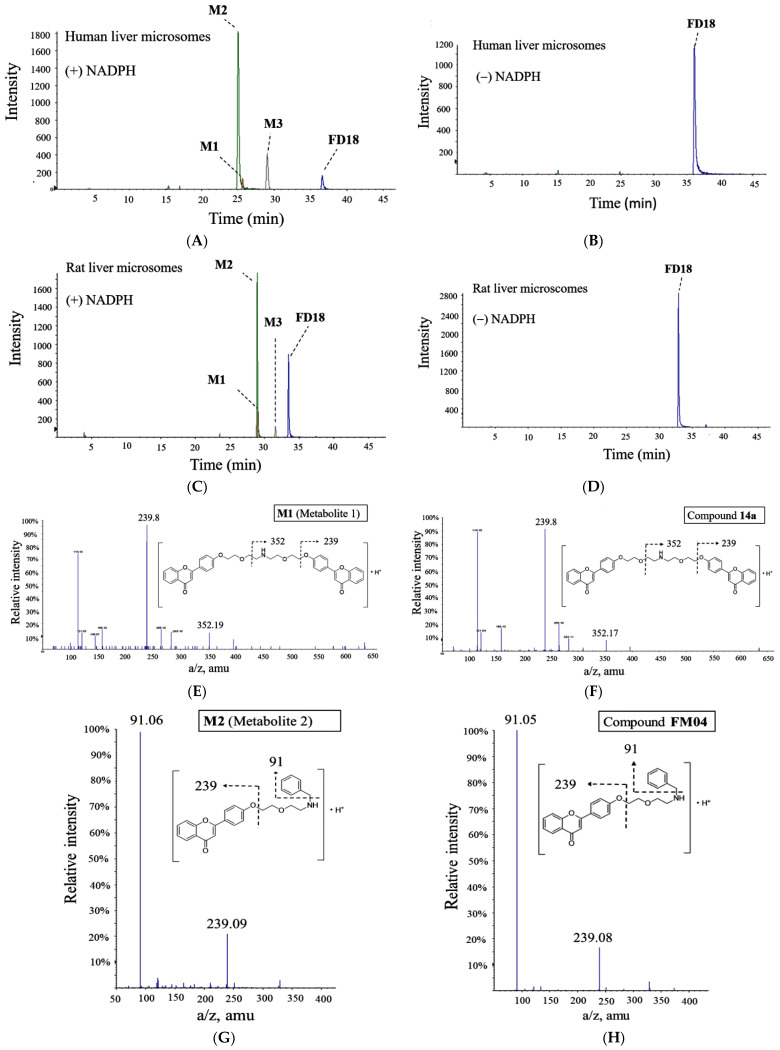

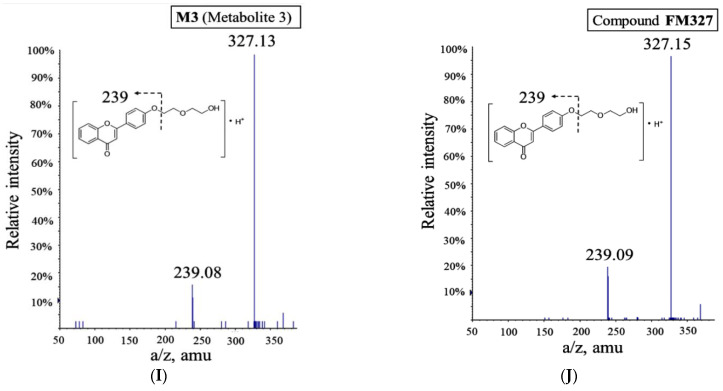
Identification of metabolites of **FD18**. LC/QTOF-MS extracted ion chromatograms of **FD18** incubated with liver microsomes. Human liver microsomes (HLM) or rat liver microsomes (RLM) were incubated with **FD18** with or without NADPH for 30 min at 37 °C. Metabolite mixture was separated by LC/QTOF-MS. Three metabolites were found after incubation. **FD18** (724 *m*/*z*); **M1** = metabolite 1 (634 *m*/*z*); **M2** = metabolite 2 (416 *m*/*z*); **M3** = metabolite 3 (327 *m*/*z*). (**A**) **FD18** and HLM (+ NADPH). (**B**) **FD18** and HLM (−NADPH). (**C**) **FD18** and RLM (+ NADPH). (**D**) **FD18** and RLM (− NDPH). Metabolites **M1, M2** and **M3** were subjected to LC/QTOF-MSMS analysis and their mass spectrums were shown here. According to the mass spectrum, the respective structure of **M1**, **M2** and **M3** were predicted and synthesized. Then, the pure synthetic compounds were analyzed by the MS. (**E**) mass spectrum of **M1**. (**F**) mass spectrum of synthetic compound **14a** which has been reported in 2012 [23]. (**G**) mass spectrum of **M2**. (**H**) mass spectrum of synthetic compound **FM04** which has been reported in 2021 (Reprinted/adapted with permission from ref. [25] copyright © 2021 by American Society for Microbiology). (**I**) mass spectrum of **M3**. (**J**) mass spectrum of synthetic compound **FM327**.

We evaluated the metabolic rate of **FD18** using human liver microsomes (HLM) and rat liver microsomes (RLM). Metabolites were identified by comparing the LC-MS (QTOF) chromatograms after incubation with or without NADPH in both HLM (Figure 2A,B) and RLM (Figure 2C,D) systems. Three identical metabolites were detected in both HLM and RLM systems. Metabolites **M1, M2** and **M3** were identified with a singly charged mass-over-charge ratio of 634 *m*/*z*, 416 *m*/*z* and 327 *m*/*z*, respectively (Figure 2A,C). In both systems, **M2** was the most abundant metabolite (Figure 2A,C).

**Figure 3:**
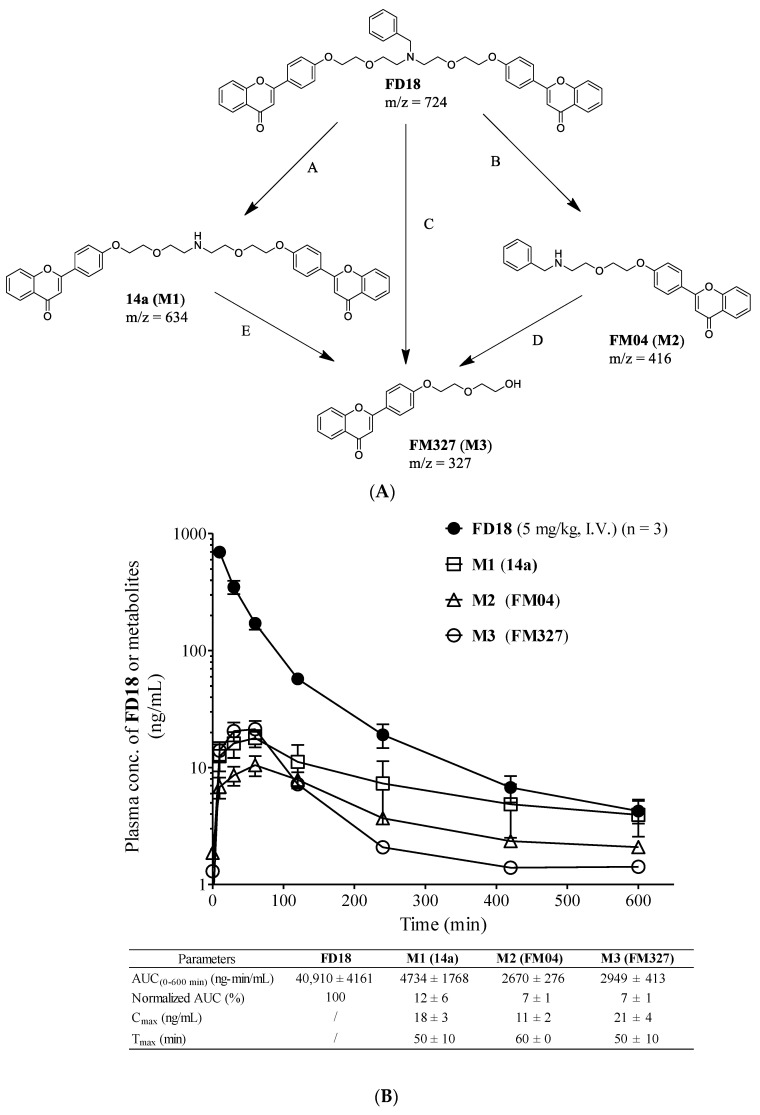
Proposed metabolism pathway of **FD18** and its metabolites formed in vivo. (**A**) metabolism study of **FD18** was conducted on human liver microsomes (HLM) and rat liver microsomes (RLM) in vitro. Chemical structure of the 3 metabolites was predicted by LC/QTOF-MS/MS and confirmed by the synthetic compounds (Figure 2E–J). The pathways for biotransformation of **FD18** into **FM04** was proposed. (**B**) plasma concentration profile of **FD18** and the in vivo formation of metabolites **M1**, **M2** and **M3.** After I.V. injection of **FD18** (5 mg/kg) into mice, plasma was collected at 10, 30, 60, 120, 240, 420 and 600 min, respectively. The concentration of **FD18** at each time point was determined by UPLC-MS/MS. Its plasma concentration was further quantified with peak area ratio of **FD18** against **D7-18**. The values were presented as mean ± standard error of mean (n = 3 mice for each time point). In vivo formation of metabolites **M1**, **M2** and **M3** were monitored and quantified by UPLC-MS/MS with corresponding calibration curve of **14a**, **FM04** and **FM327**. Pharmacokinetic parameter AUC was calculated by the pharmacokinetic software-Summit^®^ PK solutions 2.0 (Summit Research Service, Ashland, Wilmington, DE, USA).

Structures for the metabolites **M1, M2** and **M3** were proposed according to their accurate masses obtained (Figure 2A,C). They were further verified by comparing their MS/MS spectra and retention times with the authentic compounds prepared by chemical synthesis. Metabolite **M1** was identical to our previously reported flavonoid dimer compound **14a** [23] (Figure 2E,F). The structure of metabolites **M2** and **M3** was validated in tandem with mass spectrometry analysis with our synthesized compounds **FM04** (Figure 2G,H) and **FM327** (Figure 2I,J), respectively. The data suggested that rat and human liver microsomes metabolized **FD18** similarly. The metabolic pathway of **FD18** was proposed in Figure 3A. **FD18** was metabolized via the cleavage of the nitrogen-carbon bond: (A) *N*-debenzylation in the linker to form **M1** (**14a**), (B) *N*-dealkylation to form **M2** (**FM04**), (C) *N*-dealkylation to form **M3** (**FM327**). (D) **M2** can be further cleaved to form **M3** (**FM327**) or (E) **M1** was further *N*-dealkylated to form **M3** (**FM327**).

### 2.2. In Vivo Monitoring of Three Metabolites **M1**, **M2**, **M3** Formation

The in vivo formation of the three metabolites following intravenous administration (I.V.) of **FD18** was evaluated in SD rats. All three metabolites **M1**, **M2** and **M3** were detected in the plasma. The maximum plasma concentration (C_max_) of the metabolites **M1**, **M2** and **M3** were 18, 11 and 21 ng/mL between (T_max_) 50 to 60 min, respectively (Figure 3B). Systemic exposure for metabolites **M1**, **M2** and **M3** were 12%, 7% and 7%, respectively (Figure 3B).

### 2.3. In Vitro P-gp-Modulating Activity of Metabolites 14a (M1), FM04 (M2) and FM327 (M3)

The P-gp-modulating activity of the **FD18** metabolites was evaluated using authentic compounds (**14a**, **FM04** and **FM327)** in the P-gp overexpressing cell line LCCMDR. It was a multidrug-resistant cell line and showed 24- to 62-fold higher resistance towards a panel of anticancer drugs as compared to the wild-type LCC6 (Table S1). Compound **14a** (**M1,** EC_50_ = 305 nM) showed 2.1-fold lower activity than the parent **FD18** (EC_50_ = 148 nM) in reversing PTX resistance (Table 1), implying that the *N*-benzyl group in the linker is beneficial for P-gp modulation. **FM327** (**M3**) displayed no activity with EC_50_ > 1000 nM (Table 1). This is not unexpected based on our previous studies that flavonoid dimers with the appropriate linker lengths are more potent than the corresponding monomers [23]. Unlike **FM327**, **FM04** (**M2**) was a flavonoid monomer but with the *N*-benzyl group in the linker. It interestingly showed 1.8-fold higher potency than **FD18** and with EC_50_ of 83 nM for reversing PTX resistance. It also chemosensitized the LCC6MDR cells towards vinblastine, vincristine, DOX, daunorubicin and mitoxantrone with EC_50_ ranging from 61 nM to 153 nM (Table 1). At this range of concentration, **FM04** alone did not exhibit cytotoxicity towards LCC6MDR cells (Table S2). In addition, **FM04** at 1 μM did not chemosensitize the wild-type LCC6 cells towards PTX, vinblastine and vincristine, indicating that **FM04** specifically inhibits the activity of P-gp transporter (Table S1). The higher potency noted in **FM04** (**M2**) suggested that flavonoid monomer containing *N*-benzyl substituent in the linker leads to stronger inhibitory effect on P-gp than the corresponding dimeric flavonoid, contrary to our expectation [23].

**FM04** may possess better drug-like properties with a smaller molecular weight (MW of **FM04**: 415 vs. MW of **FD18**: 724), higher aqueous solubility (CLogP of **FM04**: 4.9 vs. CLogP of **FD18**: 9.0) and higher cell membrane permeability (tPSA of **FM04**: 56.8 Å^2^ vs. tPSA of **FD18**: 92.8 Å^2^) (Table 1). All in all, **FM04** with high potency and improved physicochemical properties may have promising in vivo anti-tumor efficacy.

### 2.4. Selectivity of FM04 towards P-gp, BCRP and MRP1 Transporters

**FM04** and P-gp inhibitor cyclosporine A at 1 μM lowered the IC_50_ of paclitaxel in LCC6MDR by 28- and 72-fold, respectively (Table 2). In both HE293/R2 and 2008/MRP1 cell lines, **FM04** at 1 μM did not reverse BCRP-mediated topotecan resistance or MRP1-mediated DOX resistance (Table 2). However, BCRP inhibitor Ko143 and MRP1 inhibitor **FD-4e** [26] markedly lowered the IC_50_ by around 14-fold, respectively (Table 2). Therefore, **FM04** is a mono-selective P-gp inhibitor.

### 2.5. **FM04** Increases Intracellular DOX Accumulation in LCC6MDR Cells

**Figure 4:**
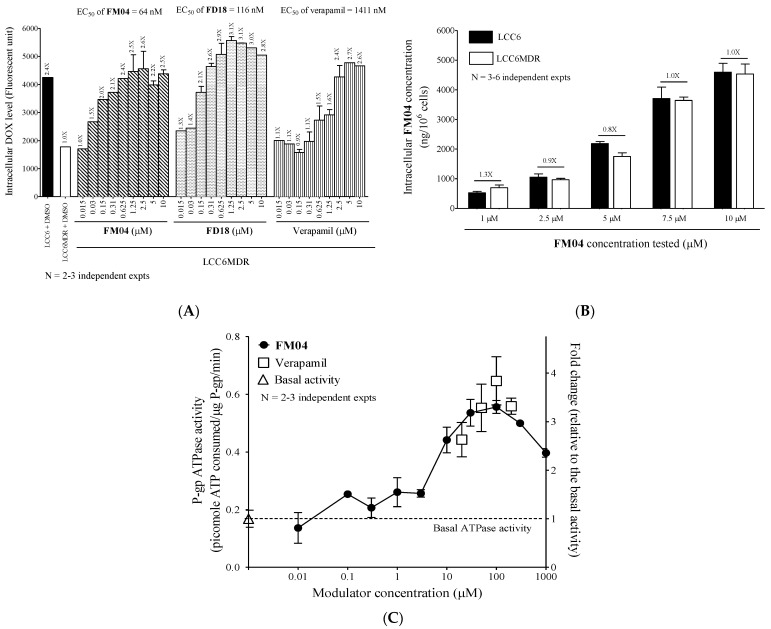
Mechanistic study of **FM04** to reverse P-gp-mediated drug resistance. (**A**) LCC6MDR cells were co-incubated with 20 μM DOX and various concentrations of modulators (0.015, 0.03, 0.15, 0.31, 0.625, 1.25, 2.5, 5 and 10 μM) including **FM04**, **FD18** and verapamil at 37 °C for 150 min. After incubation, the cells were lysed and the intracellular DOX level was determined by fluorescence microplate reader. EC_50_ (nM) was an effective concentration of modulator at which it can increase the DOX retention by 50%. The fold change of DOX concentration after each modulator treatment was relative to that of LCC6MDR treated with DMSO. The DOX levels were presented as mean ± standard error of mean. N = 2–3 independent experiments. (**B**) LCC6MDR and LCC6 cells were incubated with different concentrations of **FM04** (1, 2.5, 5, 7.5 and 10 μM) at 37 °C for 120 min. After incubation, the cells were lysed and spun down. The concentration of **FM04** in the supernatant was measured by UPLC-MS/MS. The values were presented as mean ± standard error of mean. N = 2–6 independent experiments. (**C**) membrane vesicles containing recombinant human P-gp were incubated with a series concentrations of **FM04** (0.01, 0.1, 0.3, 1, 3, 10, 70, 100, 300 and 1000 μM) and verapamil (20, 50, 100 and 200 µM) at 37 °C for 120 min. A 1% DMSO was a solvent control. After incubation, the level of the remaining ATP within the reaction system was measured through luminescence changes. The ATPase activity after each treatment was relative to the basal activity and presented as fold change. The values were presented as mean ± standard error of mean. N = 2–3 independent experiments.

The question of whether **FM04** reverses the P-gp-mediated drug resistance by increasing the intracellular drug accumulation was studied. A P-gp fluorescent substrate, DOX, was employed for studying the transport activity of P-gp and its intracellular level was measured after various P-gp inhibitor treatments. P-gp-overexpressing LCC6MDR cells accumulated 2.4-fold less DOX than its wild-type LCC6 (Figure 4A). A dose-dependent increase in DOX accumulation was observed after the LCC6MDR cells co-incubating DOX with **FM04**, **FD18** or verapamil (Figure 4A). The EC_50_ of the modulator at which it can increase the DOX retention by 50% in LCC6MDR cells was used to differentiate the potency of the modulator. The EC_50_ of **FM04** (EC_50_ = 64 nM) was 1.8-fold and 22-fold lower than that of **FD18** (EC_50_ = 116 nM) and verapamil (EC_50_ = 1411 nM), respectively (Figure 4A). Once again, monomeric **FM04** consistently showed higher potency than dimeric **FD18** not only in chemosensitizing LCC6MDR cells to various chemotherapeutic drugs (Table 1), but also in restoring the intracellular drug concentration.

### 2.6. **FM04** Is Not a Transport Substrate of P-gp

P-gp is a transporter with broad substrate specificity. To explore whether **FM04** can be pumped by P-gp and work as a competitive inhibitor to prevent the drug substrate from binding, the following experiment was performed. The intracellular level of **FM04** was compared in P-gp overexpressing LCC6MDR cells and non-P-gp parental LCC6 cells after its treatment. It was found that **FM04** was retained at a similar level in LCC6MDR and LCC6 cells at all tested concentrations (1, 2.5, 5, 7.5 and 10 µM) (Figure 4B), suggesting that **FM04** is not a transport substrate of P-gp.

### 2.7. **FM04** Does Not Inhibit P-gp ATPase Activity

The efflux activity of P-gp is driven by ATP hydrolysis. Whether **FM04** can inhibit the P-gp ATPase activity to directly block the drug efflux was investigated. Verapamil, a well-known stimulator of P-gp ATPase, can increase the P-gp ATPase activity by 2.6-fold at 20 μM to 3.3-fold at 200 μM (Figure 4C). **FM04** showed a dose-dependent manner and increased the activity from 1.5-fold at 3 μM to 3.3-fold at 100 µM (Figure 4C). This data suggested that **FM04** can induce P-gp ATPase activity after its binding to P-gp.

### 2.8. **FM04** Reverses P-gp-Mediated PTX Drug Resistance in Human Melanoma MDA435/LCC6MDR Mice Xenograft Model

We previously reported that the combination treatment of **FD18** and PTX successfully inhibited P-gp overexpressed LCC6MDR tumor growth [24]. To explore if **FM04** (metabolite **M2** of **FD18**) can modulate the PTX resistance in the LCC6MDR xenograft model, **FM04** at 28 mg/kg (I.P.) (equivalent molar concentration as **FD18** at 45 mg/kg) was co-administered with 12 mg/kg of PTX (I.V.) in treating LCC6MDR tumor.

**Figure 5:**
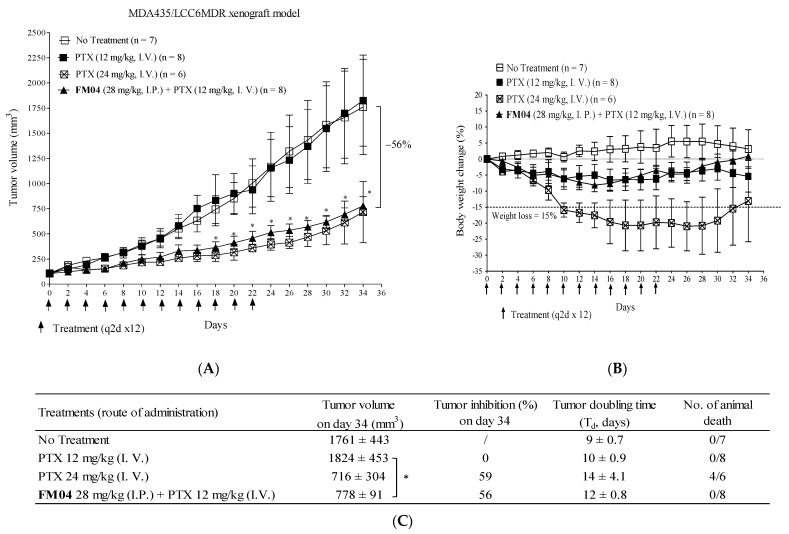
In vivo efficacy study of **FM04** on reversing P-gp-mediated PTX resistance in human melanoma MDA435/LCC6MDR xenograft model. Balb/c nude mice were subcutaneously xenografted with LCC6MDR. The treatment groups included (1) control—no treatment, (2) PTX (12 mg/kg, I.V.) alone, (3) PTX (24 mg/kg, I.V.) alone and (4) co-treatment: **FM04** (28 mg/kg, I.P.) was injected 1 hr prior to PTX (12 mg/kg, I.V.). (**A**) tumor volume and (**B**) body weight were monitored throughout the treatment. The treatment was given to mice every other day for 12 times (q2d × 12) and indicated as an arrow. The values were presented as mean ± standard error of mean (n = 6–8 mice per group). (**C**) Tumor-inhibition % on day 34 and tumor-doubling time after each treatment were determined. Number of animal deaths was monitored during the treatment. For the tumor volume at each time point, statistical analysis was conducted by one-way-ANOVA between co-treatment group and PTX alone treatment, * p < 0.05. /: not applicable.

The treatment was given every other day for 12 times (q2d × 12) from day 0 to day 22. There were four treatment groups including (1) control: no treatment, (2) PTX alone (12 mg/kg, I.V.), (3) PTX alone (24 mg/kg, I.V.) and (4) combination treatment: **FM04** (28 mg/kg, I.P.,−1 h) + PTX (12 mg/kg, I.V.). Both the control and PTX at 12 mg/kg (I.V.) groups comparatively exhibited high tumor growth rate (Figure 5A), indicating that PTX at 12 mg/kg had no inhibitory effect on tumor growth. Mice treated at a higher dosage of PTX (24 mg/kg, I.V.) resulted in a 2.5-fold reduction in tumor volume, but with severe toxicity to the mice resulting in > 15% weight loss (Figure 5B) and death (4 out of 6 mice) (Figure 5C). Combination treatment of **FM04** with PTX at 12 mg/kg significantly suppressed the tumor growth of LCC6MDR and with a 2.3-fold (*, *p* < 0.01) reduction in tumor volume (Figure 5A), clearly demonstrated that **FM04** can potentiate the anti-tumor activity of PTX. The tumor doubling time in the combination treatment group was 12 days, which was longer than that in the PTX (12 mg/kg) alone group (10 days) and control group (9 days) (Figure 5C). Furthermore, co-treatment comprised of **FM04** (28 mg/kg) together with PTX as low as 12 mg/kg successfully demonstrated promising efficacy without toxicity and animal death (Figure 5B,C).

### 2.9. Pharmacokinetics Study of PTX or **FM04** after Their Oral Co-Administration

The in vitro (Table 1) and in vivo efficacy studies (Figure 5) clearly demonstrated that **FM04** was a promising P-gp inhibitor. PTX, though widely used for the treatment of various types of cancer [27,28,29,30], is a good P-gp substrate [31]. It has low oral bioavailability because the expression of P-gp in the enterocytes within the gastrointestinal (GI) tract which actively pumps the PTX back into the gut lumen. Cancer patients have to stay in a hospital to receive the intravenous (I.V.) infusion of PTX. The I.V. treatment of PTX increases the risk of bacterial infection, the cost of treatment and makes it less comfortable and inconvenient for the patients. We hypothesized that **FM04** may be a promising compound to inhibit P-gp activity in the enterocytes within the GI tract and potentiate oral PTX bioavailability.

**Figure 6:**
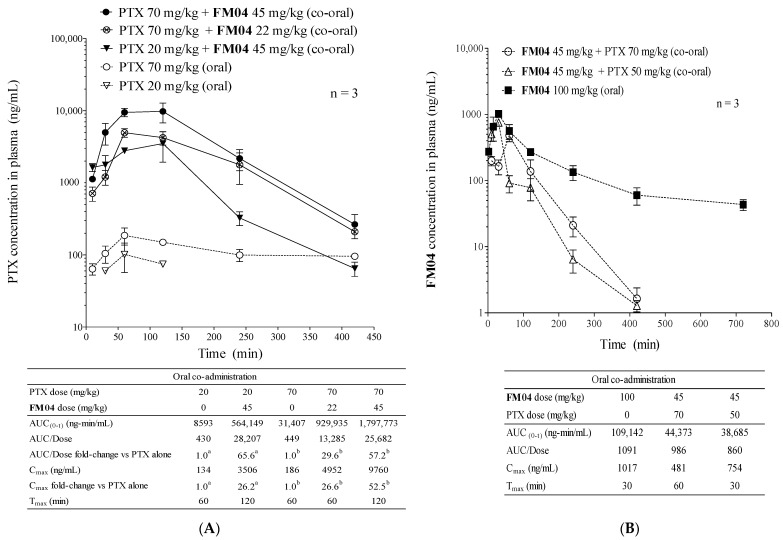
**FM04** increased oral bioavailability of PTX. Pharmacokinetics (PK) study of PTX or **FM04** after their oral co-administration. (**A**) oral PTX PK profile under the effect of **FM04** administration. PTX (20 or 70 mg/kg) was orally co-administered with **FM04** (0, 22 or 45 mg/kg) to mice, plasma was collected at 30, 60, 120, 240 and 420 min, respectively. The concentration of PTX at each time point was determined by UPLC-MS/MS. ^a^ Fold change of AUC/Dose or C_max_ was relative to the 20 mg/kg PTX alone. ^b^ Fold change of AUC/Dose or C_max_ was relative to the 70 mg/kg PTX alone. The values were presented as mean ± standard error of mean (n = 3 mice for each time point). (**B**) oral **FM04** PK profile under the effect of PTX administration. **FM04** (45 or 100 mg/kg) was orally co-administered with PTX (0, 50 or 70 mg/kg) to mice, plasma was collected at 30, 60, 120, 240 and 420 min, respectively. The concentration **FM04** at each time point was determined by UPLC-MS/MS Pharmacokinetic parameter AUC was calculated by the pharmacokinetic software-Summit^®^ PK solutions 2.0 (Summit Research Service, Ashland, Wilmington, DE, USA).

The pharmacokinetics of PTX or **FM04** after oral co-administration to mice was summarized in Figure 6A,B. With the PTX dose alone, at either 20 mg/kg (4 mg/mL) or 70 mg/kg (14 mg/mL) orally, its oral plasma AUC was 8593 and 31,407 ng/mL, respectively (Figure 6A). The oral absorption of PTX was poor at 0.2%. In the oral co-administration with **FM04** (at 45 mg/kg), the dose exposure of oral PTX was substantially enhanced as reflected by the high AUC values of 564,149 (20 mg/kg of PTX) and 1,797,773 ng/mL (70 mg/kg of PTX), respectively (Figure 6A). There were 66-fold and 57-fold improvement of AUC and the oral PTX absorption was increased from 0.2% without **FM04** to 14% with **FM04**, respectively. When **FM04** was reduced to 22 mg/kg, the AUC (929,935 ng/mL) was halved; however, it was still a 30-fold increase in AUC and with 6.6% of oral absorption as compared to PTX alone (70 mg/kg). Consistently with the AUC data, C_max_ was 26-, 27- and 53-fold dramatically increased after co-administration of **FM04** (45 mg/kg) + PTX (20 mg/kg) and **FM04** (22 mg/kg or 45 mg/kg) + PTX (70 mg/kg), respectively (Figure 6A). These data strongly demonstrated that oral co-administration of the P-gp inhibitor **FM04** would significantly enhance the oral PTX absorption to reach systemic circulation.

After a single oral administration of 100 mg/kg (20 mg/mL) of **FM04** alone, its plasma level was detected from the maximum concentration of 1017 ng/mL at 30 min to the minimum concentration of 43 ng/mL at 720 min (Figure 6B). The oral absorption of **FM04** was extremely low with <0.005 %, suggesting that its P-gp modulation occurred locally in the GI tract but not in the other organs. Moreover, PTX at 50 or 70 mg/kg did not significantly affect the AUC of **FM04** (Figure 6B) after its co-administration, implying that there is little competition for P-gp transport in the GI tract, nor drug–drug interaction between **FM04** and PTX.

### 2.10. Mechanistic Studies of **FM04** on the Enhancement of Oral Bioavailability of PTX In Vitro

The low oral bioavailability of PTX was due to its high affinity for the P-gp transporter and the activity of metabolic enzymes such as CYP2C8 and CYP3A4 in the GI tract and liver. Whether **FM04** can inhibit the P-gp located within the GI tract or alter the metabolism of PTX was studied using transepithelial permeation and liver microsome assays.

#### 2.10.1. Effect of **FM04** on Transepithelial Transport of PTX Using Caco-2 Transwell Assay In Vitro

Caco2 is a human colon cancer cell line and its polarized monolayer, like the intestinal epithelial barrier, is widely used for small molecule intestinal permeation in vitro studies. In the permeation assay, the compound can be added at either apical or basal chambers after Caco2 cells form a monolayer on the transwell. The transport of drug molecules between the two chambers can be evaluated. From apical to basolateral transport (A to B, mimics drug uptake into the systemic circulation), the compound was added at the apical side and samples of the medium were collected at the basal side and vice versa for basal to apical transport (B to A, mimics drug efflux to the gut lumen).

**Figure 7:**
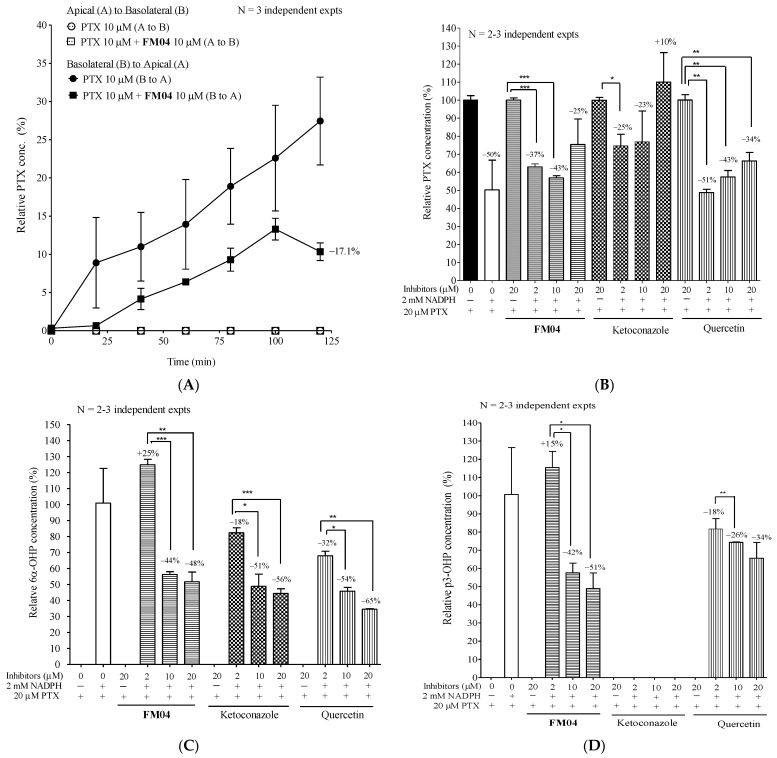
Mechanistic study of **FM04** to increase the oral PTX absorption. (**A**) transepithelial permeability of PTX in Caco-2 transwell assay. When confluent monolayer of Caco2 cells was formed on the transwell, the permeability of PTX (10 μM) was studied with or without **FM04** (10 μM). For apical to basolateral transport (A to B), compound was added at the apical side (donor) and samples of the medium collected at the basal side (receiver) at 0, 20, 40, 60, 80, 100 and 120 min and vice versa for basal to apical transport (B to A). The PTX level was determined using UPLC-MS/MS. The relative % of PTX was presented as mean ± standard error of mean. N = 2–3 independent experiments. Impact of **FM04** on PTX metabolism. Ketoconazole and quercetin, known inhibitors of CYP2C8 or 3A4 metabolic enzymes, were included in the assay. PTX at 20 μM was incubated with a series concentration of **FM04**, ketoconazole or quercetin (2, 10 and 20 μM) in human liver microsomes with or without NADPH, respectively. After incubation, the levels of PTX, metabolites 6α-hydroxpaclitaxel (6α-OHP) and p3′-hydroxpaclitaxel (p3-OHP) were determined by HPLC-MS/MS. (**B**) changes in PTX. (**C**) changes in 6α-OHP. (**D**) changes in p3-OHP. Relative concentration (%) of PTX, 6α-OHP and p3-OHP were presented as mean ± standard error of mean. N = 2–3 independent experiments. Statistical analysis was performed by using Student’s t-test, * *p* < 0.05; ** *p* < 0.01; *** *p* < 0.001.

Without the P-gp modulator **FM04**, PTX at 10 µM was only pumped from B to A, but not A to B (Figure 7A), indicating that Caco2 cells on the transwell did not uptake PTX, but they extruded PTX. It also revealed that the active transporter P-gp was expressed in the Caco2 cells. **FM04** at 10 μM diminished the PTX transport from B to A by 17%, respectively (Figure 7A). This decrease in B to A PTX permeability following P-gp inhibition by **FM04** highly supports the PK data that the oral co-administration with **FM04** remarkably enhances the oral PTX plasma level by inhibiting the P-gp-mediated PTX efflux into the gut lumen (Figure 6A).

#### 2.10.2. Effect of **FM04** on PTX Metabolism Using Human Liver Microsomes Assay In Vitro

PTX metabolism was studied using human liver microsomes with or without NADPH. Ketoconazole and quercetin are typical inhibitors of CYP2C8 and CYP3A4 which are the major enzymes for the metabolic transformation of PTX in humans. The effect of **FM04** and CYP inhibitors on the levels of PTX (Figure 7B), its metabolites 6α-hydroxypaclitaxel (6α-OHP) (Figure 7C) and p-3′-hydroxypaclitaxel (p3-OHP) (Figure 7D) were investigated. In the absence of NADPH, no PTX metabolism occurred in 0 or 20 μM inhibitor treatments (Figure 7B). In the presence of NADPH, PTX level was reduced by 50% (Figure 7B). The addition of **FM04**, ketoconazole or quercetin significantly inhibited the PTX metabolism because a relatively lower amount of PTX was consumed at 2, 10 and 20 μM when tested (Figure 7B). PTX metabolite 6α-OHP is a product of CYP2C8 activity and it was solely detected in the presence of NADPH (Figure 7C). P-gp inhibitor **FM04** at 2 µM, did not inhibit the CYP2C8 activity because the high level of 6α-OHP was detected. However, at 10 or 20 μM of **FM04**, the level of 6α-OHP was significantly diminished by 44 or 48% (Figure 7C), suggesting that CYP2C8 activity was inhibited by **FM04**. A dose-dependent inhibition of CYP2C8 enzyme activity was also noted when the samples were treated with ketoconazole and quercetin (Figure 7C). Enzymatic activity of CYP3A4 in the presence of NADPH resulted in the production of PTX metabolite p3-OHP (Figure 7D). **FM04** did inhibit the CYP3A4 activity because there was a 42 or 51% reduction in p3-OHP when the sample was treated with 10 or 20 µM of **FM04** (Figure 7D). A drop in p3-OHP level was also noted after quercetin treatment. Ketoconazole, a strong CYP3A4 inhibitor, did not result in any p3-OHP production, indicating that there was complete inhibition of CYP3A4 enzymes (Figure 7D) even at 2 µM.

After 10 or 20 µM **FM04** treatment, the decreased levels of both metabolites 6α-OHP (Figure 7C) and p3-OHP (Figure 7D) were in good agreement with the decreased PTX metabolism (Figure 7B). **FM04** was clearly proved to inhibit both the active transporter P-gp present in the intestinal Caco2 cells and the PTX metabolic enzymes CYP2C8 and CYP3A4; consistent with the observation that it potentiated the oral PTX bioavailability (Figure 6A).

### 2.11. Efficacy of Orally Co-Administered **FM04** and PTX in Treating the Human Melanoma MDA435/LCC6 Xenograft Model In Vivo

**Figure 8:**
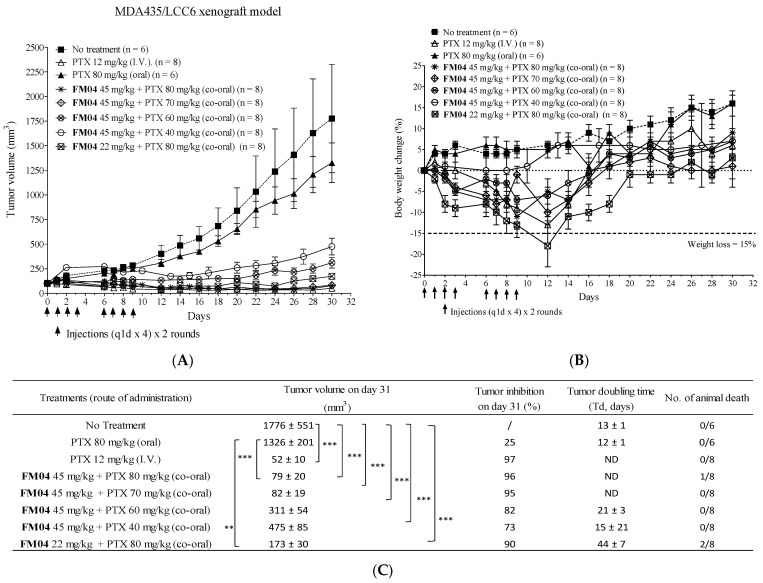
In vivo anti-tumor efficacy of oral co-administration of **FM04** with PTX in human melanoma MDA435/LCC6 xenograft. Balb/c nude mice were subcutaneously xenografted with LCC6. The treatment groups included (1) control: no treatment, (2) PTX 12 mg/kg (I.V.) alone, (3) PTX 80 mg/kg (oral) alone, (4) co-treatment: **FM04** 45 mg/kg + PTX 80 mg/kg (co-oral), (5) co-treatment: **FM04** 45 mg/kg + PTX 70 mg/kg (co-oral), (6) co-treatment: **FM04** 45 mg/kg + PTX 60 mg/kg (co-oral), (7) co-treatment: **FM04** 45 mg/kg + PTX 40 mg/kg (co-oral) and (8) co-treatment: **FM04** 22 mg/kg + PTX 80 mg/kg (co-oral). (**A**) Tumor volume and (**B**) body weight were monitored throughout the treatment. The treatment was given to mice every day for 4 times and 2 rounds [(q1d × 4) x 2 rounds] and indicated as an arrow. The values were presented as mean ± standard error of mean (n = 6–8 mice per group). (**C**) Tumor inhibition % on day 30 and tumor doubling time after each treatment were determined. Number of animal deaths was monitored during the treatment. ND = tumor-doubling time of co-treatment groups **FM04** at 45 mg/kg + PTX at 80 or 70 mg/kg or PTX alone at 12 mg/kg (I.V.) cannot be determined as tumor volume was smaller than the initial tumor volume 100 mm^3^. For the tumor volume at each time point, statistical analysis was conducted by one-way-ANOVA between co-treatment group and control or PTX alone treatment. ** *p* < 0.01; *** *p* < 0.001. /: not applicable.

Next, we evaluated if oral co-administration of **FM04** with PTX can effectively inhibit tumor growth in vivo. Here, we employed the human melanoma LCC6 xenograft model for the in vivo anti-tumor efficacy study and the effect of oral co-treatment on tumor volume was summarized in Figure 8A. There were eight treatment groups including (1) control: no treatment; (2) PTX 12 mg/kg, I.V.; (3) PTX 80 mg/kg, oral; (4) **FM04** 45 mg/kg + PTX 80 mg/kg, co-oral; (5) **FM04** 45 mg/kg + PTX 70 mg/kg, co-oral; (6) **FM04** 45 mg/kg + PTX 60 mg/kg, co-oral; (7) **FM04** 45 mg/kg + PTX 40 mg/kg, co-oral; and (8) **FM04** 22 mg/kg + PTX 80 mg/kg, co-oral. The treatment was given daily four times and two rounds on days 0, 1, 2, 3, 6, 7, 8 and 9 [(q1d × 4) × 2 rounds].

Tumor volume after oral dosing with PTX (80 mg/kg) alone did not produce significant tumor suppression (Figure 8A). In contrast, intravenous administration of PTX (12 mg/kg) alone significantly caused suppressive tumor growth with 97% (***, *p* < 0.001) inhibition as compared to the non-treatment control. Oral co-treatment of **FM04** (45 mg/kg) with PTX (40, 60, 70 or 80 mg/kg) resulted in promising treatment outcomes with 73% (***, *p* < 0.001), 82% (***, *p* < 0.001), 95% (***, *p* < 0.001) and 96% (***, *p* < 0.001) tumor growth inhibition on day 30, respectively (Figure 8A). When half amount of **FM04** (22 mg/kg) was combined with PTX (80 mg/kg), it still exhibited a strong anti-tumor effect with 90% (***, *p* < 0.001) inhibition (Figure 8A).

These data clearly demonstrated that **FM04** can overcome the unresponsiveness from the oral-dosed PTX in vivo because **FM04** inhibited both active transporter P-gp in the GI tract (Figure 7A) and PTX metabolic CYP enzymes (Figure 7B–D) to boost the oral PTX bioavailability and eventually PTX achieved its plasma therapeutic level to kill the tumor cells. Oral co-administration of **FM04** (45 mg/kg) + PTX (70 or 80 mg/kg) caused highly comparable tumor inhibition to that of I.V. administration of PTX (12 mg/kg) alone (Figure 8A). The tumor-doubling time of co-treatment groups was extended to 15–44 days as compared to the no-treatment or oral PTX-alone groups (12–13 days) (Figure 8C). However, the tumor-doubling time of effective co-treatment groups **FM04** at 45 mg/kg + PTX at 70 or 80 mg/kg cannot be determined because the tumor size was smaller than the 100 mm^3^ (the initial tumor volume before treatment) during the treatment period (Figure 8A).

Treatment-induced toxicity was observed in certain co-treatment groups, although it did not reflect in severe body weight loss (> 15% loss in 3 consecutive days) (Figure 8B). Animal death was observed in co-treatment (**FM04** 45 mg/kg + PTX 80 mg/kg) and (**FM04** 22 mg/kg + PTX 80 mg/kg) groups (Figure 8C). This suggests that the oral PTX dosage in the presence of **FM04** at 45 or 22 mg/kg should not exceed 70 mg/kg.

## 3. Discussion

Flavonoid dimer **FD18,** with adequate potency and acceptable safety, was demonstrated to significantly reverse the P-gp-mediated MDR in tumor-bearing mice [24]. To further assess the safety and intrinsic properties of **FD18**, its metabolism and pharmacokinetics were investigated. In the liver microsome study, **FD18** was found to be metabolized into **14a**, **FM327** and **FM04** (Figure 2A,C). Interestingly, metabolite **FM04**, a flavonoid monomer with *N*-benzyl substituent in the linker, exhibited potent P-gp modulating activity (1.8-fold higher than the parent dimeric **FD18**) in reversing PTX resistance and increasing intracellular DOX accumulation in LCC6MDR cells (Table 1 and Figure 4A).

Similar to the parent **FD18**, **FM04** modulated the P-gp-mediated drug resistance by inhibiting the transport function of P-gp and then restoring the intracellular drug concentration to the wild-type level (Figure 4A). Both were stimulators of P-gp ATPase (Figure 4C) and did not directly inhibit the drug efflux. While **FD18** was found to be a substrate of P-gp and transported out of the LCC6MDR cells [23], **FM04** itself was not a transport substrate of P-gp (Figure 4B). Moreover, it also exhibited similar cytotoxicity towards LCC6 and LCC6MDR (Table S2). It is likely that **FM04** is an allosteric modulator rather than a competitive inhibitor. Using in silico molecular docking studies, it was proposed that apigenin may bind to the NBDs of P-gp [32]. It has been proposed that ATP binding and hydrolysis at NBD domains can control the substrate binding and release by regulating the “high-affinity conformation” and “low-affinity conformation” of P-gp for the substrates [33]. **FM04** might stabilize the conformation of P-gp after its binding and then change the binding affinity for the substrate.

Metabolite **FM04** is more druggable than **FD18** to be further developed as a novel combination chemotherapy because of its (1) higher potency (EC_50_ of **FM04**: 64 to 83 nM vs. EC_50_ of **FD18**: 116 to 148 nM), (2) smaller molecular weight (MW of **FM04**: 415 vs. MW of **FD18**: 724), (3) higher aqueous solubility (CLogP of **FM04**: 4.9 vs. CLogP of **FD18**: 9.0) and (4) higher cell membrane permeability (tPSA of **FM04**: 56.8 Å^2^ vs. tPSA of **FD18**: 92.8 Å^2^) (Table 1 and Figure 4A). The increased hydrophilicity of **FM04** allows it to be formulated in 5% ethanol whereas **FD18** was formulated in 5% Cremophor EL (CrEL) and 5% ethanol. CrEL is a vehicle for solubilization of some commercially available hydrophobic drugs, but it has the drawbacks of causing serious hypersensitivity reactions and neurological toxicity in patients [34]. Moreover, it can alter the disposition and pharmacodynamics of some drugs [35,36]. Therefore, **FM04** has a potential therapeutic advantage over **FD18** when it is solubilized in a CrEL-free formulation.

Without **FM04,** PTX at 12 mg/kg (I.V.) had no inhibitory effect on P-gp-overexpressed LCC6MDR tumor growth (Figure 5A). Co-treatment of **FM04** (I.P.) with PTX at 12 mg/kg (I.V.) given to LCC6MDR tumor-bearing mice resulted in 56% tumor volume reduction (*, *p* < 0.05) (Figure 5A). No animal death or > 10% body weight loss were noted in the co-treatment group (Figure 5B,C). Thus, **FM04** did not enhance the toxicity of PTX. This result illustrates the safety advantage of using co-treatment of **FM04** and PTX to modulate the P-gp-mediated PTX resistance in tumor.

PTX is effective for treating various types of cancers [27,28,29,30] and is routinely applied intravenously to cancer patients [37]. Intravenous chemotherapy needs hospitalization during infusion. It increases the risk of bacterial infection and decreases the patient quality of life. Oral medication is more economical and convenient for patients. However, PTX is limited by its poor aqueous solubility and low oral bioavailability. After the oral administration of PTX, the drug is actively pumped out by P-gp and metabolized by the CYP2C8 and CYP3A4 enzymes located in the intestine, eventually causing its low intestinal uptake and systemic exposure [30]. Various approaches have been employed to enhance oral bioavailability of drugs including the use of P-gp inhibitors (e.g., cyclosporine A, valspodar SDZPSC833 or HM30181) [38,39,40] to increase oral absorption at the GI tract; the use of nanotechnology/nanoemulsion/liposome to enhance penetration [41]; or the use of CYP3A4 inhibitor (e.g., ritonavir) to reduce the drug metabolism [42].

It has been reported that the oral plasma AUC of PTX was markedly increased by 12.4-, 1.5- and 27.6-fold in P-gp KO (Mdr1a/b^−/−^), Cyp3a KO (Cyp3a^−/−^) and combined P-gp and Cyp3a KO mice (Cyp3a/Mdr1a/b^−/−^) relative to wild type mice after an oral dose of PTX at 10 mg/kg, respectively [43]. The highest AUC was found in the Cyp3a/Mdr1a/b^−/−^ mice, suggesting that both intestinal P-gp and the CYP3A enzyme work synergistically to regulate the oral PTX bioavailability. A promising strategy to boost the oral bioavailability of PTX is to combine the oral formulation of PTX with a dual inhibitor of P-gp and CYP metabolic enzymes. **FM04** was demonstrated to inhibit P-gp of intestinal Caco2 cells to export PTX from basal to apical side (Figure 7A) and decrease the PTX metabolism by inhibiting the CYP2C8 and CYP3A4 enzymes in liver microsomes (Figure 7B–D). The oral PTX absorption was significantly increased from 0.2% without **FM04** to 14% with **FM04** and there was about 30- to 66-fold improvement of oral plasma AUC of PTX after its oral co-administration with **FM04** (Figure 6A). The improved AUC value was favorably comparable to that after a complete knockout of both P-gp and CYP3A (AUC fold change = 27.6) [43], implying that a complete inhibition of both intestinal P-gp and CYP2C8 or 3A4 enzymes in mice was substantially achieved by the dual target inhibitor **FM04**.

The combined treatment of oral PTX with P-gp inhibitor cyclosporine analog SDZPSC833 resulted in an approximately 10-fold increase in the AUC of PTX [39]. In addition, oral co-administration with the P-gp inhibitor elacridar or CYP3A inhibitor ritonavir have also been reported to strongly enhance the AUC of PTX by 10.7-fold and 2.5-fold as compared to a single oral dose of PTX [42]. In agreement with the P-gp KO or CYP3A KO mice, the AUC improvement was relatively lower when co-administered with the CYP3A inhibitor than the P-gp inhibitor. Combined administration of elacridar and ritonavir was effective and gave 31.7-fold enhancement in AUC [42]. Comparatively, **FM04** (AUC fold change = 30 to 66, Figure 6A) is more efficient and superior to PDSZPSC833, elacridar or ritonabvir alone or combined elacridar and ritonabvir to be applied in oral PTX formulation.

PK (Figure 6A) and in vivo efficacy studies (Figure 8A) provided clear evidence to support PTX to become an oral agent after formulating with **FM04**. The oral co-administration (**FM04** at 22 or 45 mg/kg + PTX at 40, 60 or 70 mg/kg) displayed an encouraging therapeutic response in the human melanoma LCC6 nude mice xenograft (Figure 8A). The anti-tumor efficacy of some oral co-treatment groups (**FM04** + PTX at 70 mg/kg) were equally effective as the I.V. administration of PTX alone (Figure 8A). It has been reported that the inhibition of intestinal efflux transporters and /or CYP enzymes would cause high exposure of drug substrates in plasma and tissue and finally lead to toxicity [44]. In our study, treatment with a higher oral dosage of PTX at 80 mg/kg together with **FM04** at 22 or 45 mg/kg showed severe toxic effect with one to two animal deaths observed and weight loss in the surviving animals (Figure 8B,C). When the dosage of PTX was lowered to 70 mg/kg or less, no animal death or > 10% body weight loss were noted in that co-treatment group (Figure 8B,C). Therefore, careful adjustment of the therapeutic dosage of inhibitor and anticancer drugs will be needed in any clinical study.

The discovery of **FM04** as a potent inhibitor of P-gp poses an intriguing question: mechanistically, how does **FM04** function as an inhibitor? Both **FD18** and **FM04** can stimulate P-gp ATPase (Figure 4C) and do not directly inhibit drug efflux. While **FD18** has been found to be a substrate of P-gp [23], **FM04** itself is not a transport substrate of P-gp (Figure 4B). **FM04** therefore cannot work as a competitive inhibitor to prevent the drugs from binding to P-gp. The mechanism of action of **FM04** to modulate the P-gp-mediated drug resistance remains unclear and is under active investigation.

## 4. Materials and Methods

### 4.1. Chemicals and Reagents

Doxorubicin (DOX), daunorubicin, paclitaxel (PTX), vinblastine, vincristine and mitoxantrone were purchased from Sigma. 3-(4,5-Dimethylthiazol-2-yl)-5-[3-(carboxy- methoxy)phenyl]-2-(4-sulfo-phenyl)-2H-tetrazolium (MTS) and phenazine methosulfate (PMS) were purchased from Promega. Dulbecco’s Modified Eagle’s Medium (DMEM), Roswell Park Memorial Institute (RPMI) 1640 Medium, penicillin/streptomycin and trypsin-EDTA were purchased from Gibco BRL. Fetal bovine serum (FBS) was purchased from Hyclone Laboratories. Human melanoma cell lines MDA435/LCC6 and MDA435/LCC6MDR were kindly provided by Dr. Robert Clarke (Georgetown University, Washington, DC, USA). Human ovarian cancer cell line, 2008/MRP1, was a generous gift of Piet Borst (The Netherlands Cancer Institute, Amsterdam, The Netherlands). Human embryonic kidney cell line HEK293/R2 which was a BCRP transfectant was kindly provided by Kenneth To (The Chinese University of Hong Kong, Hong Kong, HKSAR). Caco-2 cell line was kindly provided by Thomas Leung (The Hong Kong Polytechnic University, Hong Kong, HKSAR). The mouse fibroblast cell line, L929, was purchased from ATCC. 

### 4.2. Liver Microsomes Metabolism Study of **FD18**

CYP M-class 50-donor pooled human liver microsomes (HLM), rat liver microsomes (RLM) and NADPH reagent were purchased from Research Institute for Liver Diseases (Shanghai). **FD18** (1 mg/mL in methanol) was added to 100 μL of liver microsomes at a final concentration of 20 μM. Methanol content for each reaction was less than 0.25% to avoid metabolism interference [45]. Milli-Q water was added to reach the reaction volume of 200 μL. After pre-incubating at 37 °C for 3 min, 4 μL of NADPH (final concentration of 2 mM in the reaction) was added to initiate the reaction. The reaction mixture was incubated at 37 °C for 30 min. The reaction was terminated by adding 1.5 mL of ether and then vortexed vigorously. The upper layer of the organic phase was saved and dried under mild heating at 60 °C. The extract was reconstituted in 100 μL of methanol and filtered through the 0.22 μm syringe filter before LC/QTOF-MS analysis.

### 4.3. HPLC-MS/MS Quadrupole-Time of Flight Analysis for Metabolite Identification

The LC/QTOF-MS system consisted of a Perkin Elmer Series 200 HPLC interfaced with Applied Biosystems PE SCIEX/API QSTAR Pulsar Hybrid Quadrupole-TOF mass spectrometer equipped with an electrospray ionization source in positive mode. Zorbax Eclipse XDB-C8 (4.6 × 150 mm, 5 µM) was used for separation. The mobile phase for chromatographic separation consisted of methanol + 0.1% formic acid (solvent B) and Milli-Q water + 0.1% formic acid (solvent A). The flow rate was 0.3 mL/min. The initial condition was 90% solvent A and 10% solvent B. After 3 min, a linear gradient was applied with solvent B increasing from 10% to 100% in 65 min. Solvent B at 100% was then applied for 5 min to wash out the remaining elutes. Afterwards, the mobile phase was restored to the initial condition for re-equilibration. A sample volume of 5 µL was injected for each analysis. MS detection for the full scan LC/QTOF-MS and LC/QTOF-MSMS were done in the same instrument, the mass range of m/z 50–1900 was set for LC/QTOF-MS. The settings for LC/QTOF-MS were as follows, curtain gas, 30; gas 1, 30; gas 2, 80; desolvation temperature, 350 °C; ionization voltage, 5.5 Kv. The collision energy used for metabolite identification by LC/QTOF-MSMS ranged from 18 to 55 eV; nitrogen was used as the collision gas. Data were processed by Analyst QS 1.1.

### 4.4. Synthesis of 1,13-Bis[4′-(4H-chromen-4-on-2-yl)phenyl]-1,4,10,13-tetraoxa-7-azatridecane (**14a**)

Synthesis of **14a** had been reported previously [23].

### 4.5. Synthesis of 2-(4-(2-(2-(benzylamino)ethoxy)ethoxy)phenyl)-4H-chromen-4-one (**FM04**)

Synthesis of **FM04** had been reported previously [25].

### 4.6. Synthesis of 2-(4-(2-(2-hydroxyethoxy)ethoxy)phenyl)-4H-chromen-4-one (**FM327**)

A well stirred mixture of 4′-hydroxyflavone (0.24 g, 1.0 mmol), 2-(2-chloroethoxy)ethanol (0.18 g, 1.5 mmol) and K_2_CO_3_ (0.20 g, 1.4 mmol) in ACN (20 mL) was heated to reflux for 4 hr. After that, the reaction mixture was filtered and the obtained filtrate was evaporated under reduced pressure to give pale brown oil which was subjected to flash chromatography on silica gel with gradient elution (20% acetone in DCM to 50% acetone in DCM) to furnish **327** (0.12 g, 0.37 mmol) in 37% yield: ^1^H NMR (400 MHz, CHLOROFORM-d) δ 8.23 (d, *J* = 8.31 Hz, 1H), 7.88 (d, *J* = 8.80 Hz, 2H), 7.66–7.79 (m, 1H), 7.55 (d, *J* = 8.31 Hz, 1H), 7.40 (t, *J* = 7.58 Hz, 1H), 7.05 (d, *J* = 8.80 Hz, 2H), 6.74 (s, 1H), 4.22 (t, *J* = 4.4 Hz, 2H), 3.90 (t, *J* = 4.4 Hz, 2H), 3.80 (t, *J* = 4.2 Hz, 2H), 3.71 (t, *J* = 5.14 Hz, 2H), 2.47 (br, 1H); ^13^C NMR (101 MHz, CHLOROFORM-d) δ 178.4, 163.3, 161.5, 156.2, 133.6, 128.0, 125.6, 125.1, 124.3, 123.9, 117.9, 115.0, 106.2, 72.7, 69.5, 67.6, 61.7; LRMS (ESI) *m/z* 327 (M^+^ + H, 100); HRMS (ESI) calcd for C_19_H_19_O_5_ (M^+^ + H) 327.1232, found 327.1233.


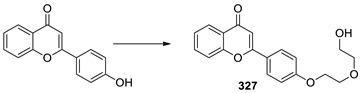


### 4.7. Cell Culture

MDA435/LCC6, MDA435/LCC6MDR, L929 and CaCo-2 cells were cultured in supplemented DMEM medium with 10% FBS, 100 U/mL penicillin and 100 μg/mL streptomycin. Then, 2008/MRP1 and HEK293/R2 cells were cultured in a supplemented RPM1640 medium with 10% FBS, 100 U/mL penicillin and 100 μg/mL streptomycin. They were incubated at 37 ℃ in a humidified atmosphere of 5% CO_2_. They were split constantly after a confluent monolayer has been formed. To split cells, the plate was washed briefly with phosphate-buffered saline (PBS), treated with 0.05% trypsin-EDTA and harvested by centrifugation.

### 4.8. In Vitro P-gp-Modulating Activity of FD18, 14a (M1), FM04 (M2) and FM327 (M3)

To each well of a 96-well plate, 6500 cells of LCC6MDR were seeded in 180 μL supplemented medium. A 10 µL compound (**FD18**, **14a**, **FM04** or **FM327**) at different concentrations (0, 62.5, 125, 250, 500 and 1000 nM) was added to the well. Various doses of PTX, DOX, daunorubicin, mitoxantrone, vinblastine or vincristine in a 10 µL volume were also added into the designated well. The final volume in each well was 200 µL and the 96-well plate was incubated at 37 °C for 5 days. The cell viability was determined using the MTS assay as reported previously [26]. The % of cell survival, IC_50_ of anticancer drugs towards the LCC6MDR and the EC_50_ of modulators were analyzed using nonlinear regression in Prism 5.0 (Prism version 5.0, GraphPad software, San Diego, CA, USA).

### 4.9. In Vitro BCRP- and MRP-1-Modulating Activity of **FM04**

To each well of the 96-well plate, 5000 cells of HEK293/R2 were incubated with different concentrations of topotecan (0, 8, 25, 74, 222, 667 and 2000 nM) with or without 1μM of **FM04** or Ko143. Then, 4000 cells of 2008/MRP1 were incubated with different doses of DOX (0, 8, 25, 74, 222, 667 and 2000 nM) with or without 1 μM of **FM04** or **FD-4e**. The final volume in each well of the 96-well plate was 200 μL. The plate was then incubated for 5 days at 37 °C. The cell viability was determined using an MTS assay as reported previously [26].

### 4.10. Cytotoxicity Assay of **FM04**

To each well of 96-well plate, 10,000 cells of LCC6, LCC6MDR or L929 were incubated with different concentrations of **FM04** (0, 0.4, 1.2, 3.7, 11, 33 and 100 μM). The final volume in each well of 96-well plate was 100 μL and the plate was incubated for 3 days at 37 °C. The cell viability was determined using MTS assay as reported previously [26].

### 4.11. Intracellular DOX Accumulation Assay

DOX accumulation assay was carried out according to the reported procedures [27]. Briefly, 1 × 10^6^ cells of LCC6 and LCC6MDR cells were incubated with different concentrations of the modulator (0, 0.015, 0.030, 0.15, 0.31, 0.625, 1.25, 2.5, 5, 10 μM) and 20 μM DOX for 150 min at 37 °C. DMSO (0.5%) was used as a negative control. After incubation, cells were washed, lysed with a lysis buffer (0.75 M HCl, 0.2% Triton-X100 in isopropanol) and the fluorescence level of DOX determined by a fluorescence microplate reader (excite at 460 nm and emits at 610 nm).

### 4.12. P-gp-ATPase Activity

P-gp-ATPase activity was measured by a P-gp-Glo assay system (Promega) with a human P-gp membrane, as reported previously [21]. The human P-gp membrane was incubated with or without 100 µM sodium vanadate containing 0.1% DMSO, verapamil (20, 50, 100 and 200 µM) and **FM04** (0.01, 0.1, 0.3, 1, 3, 10, 30, 100, 300 and 1000 µM). The reaction was initiated by the addition of MgATP and incubated at 37 °C for 1 h. After adding the ATP detection reagent, the sample was incubated at room temperature for 20 min before measuring the remaining ATP signal of luminescence by a Clariostar microplate reader (BMG Labtech). Vanadate-inhibitable P-gp ATPase activity was determined by calculating the luminescence signal in the samples with or without sodium vanadate.

### 4.13. Animal Study

Male SD rats, Balb/c or Balb/c nude mice were obtained from Centralized Animal Facilities (CAF) of the Hong Kong Polytechnic University, Hong Kong. All animals were housed in a germ-free controlled environment with a 12 h of a light and 12 h of a dark cycle with food and tap water allowed *ad libitum*. All investigations were performed following the Cap 340 Animal License from the Department of Health (HKSAR Government) and ethical approval from the Animal Ethics Sub-committee of the Hong Kong Polytechnic University. Food was removed 8–10 h before the experiment.

### 4.14. In Vivo Metabolism Monitoring of **FD18** and Its Three Metabolites **M1** (**14a**), **M2** (**FM04**), **M3** (**FM327**)

**FD18** stock solution was freshly prepared in 33% Cremophor EL, 33% ethanol and 33% saline at a concentration of 9.5 mg/mL. **FD18** stock solution was then diluted with saline to form a 4 mg/mL working solution for animal injection. SD rats were intravenously (I.V.) administered with **FD18** at 5 mg/kg (n = 3). Blood samples (approx. 300 μL) were taken via jugular vein at 10, 30, 60, 120, 240 and 420 min post-administration. Blood samples were centrifuged at 16,100× *g* for 10 min to obtain plasma. Plasma samples (50 µL) were spiked with 15 μL of internal standard (IS) (**D7-NBn**, 10 μg/mL). Two hundred microliter of methanol was added for protein precipitation. Samples were centrifuged at 16,100× *g* for 10 min prior to filtering by 0.22 μm syringe filter for UPLC-MSMS analysis.

The UPLC-MS/MS system consists of an Agilent 1260 infinity UPLC interfaced with a triple quadrupole mass spectrometer (Agilent 6460) equipped with an electrospray ionization source in positive mode. Zorbax Eclipse XDB-C8 (4.6 × 150 mm, 5 µM) was used for separation. The mobile phase for chromatographic separation consisted of methanol + 0.1% formic acid (solvent B) and Milli-Q water + 0.1% formic acid (solvent A) with gradient elution of 30% solvent B to 100% solvent B in 5 min. The elution condition was allowed for equilibrium from the 7th min onward. The analysis time for each injection was 13 min. The flow rate was 0.4 mL/min. The synthetic compound **D7-NBn**, the deuterated benzyl form of **FD18**, was used as the internal standard (IS) for the quantification of the three metabolites. Multiple reaction monitoring (MRM) was set to monitor the transitions of **D7-NBn** [M+H]^+^, **FD18** [M+H]^+^, **M1** [M+H]^+^, **M2** [M+H]^+^ and **M3** [M+H]^+^ at 731.01 > 98.1 *m*/*z*, 724.01 > 91.1 *m*/*z*, 634.01 > 114 *m*/*z*, 416.01 > 91.1 *m*/*z* and 327.01 > 239 *m*/*z*, respectively. The coefficient of determination (R^2^) for **14a, FM04** and **FM327** were above 0.99. The dynamic range for the metabolites in the current setting is 78 ng/mL to 2.44 ng/mL.

### 4.15. In Vivo Efficacy of **FM04** in Reversing PTX Resistance in the Human Melanoma MDA435/LCC6MDR Xenograft Model

The LCC6MDR tumor xenograft model was studied previously in [24]. Female Balb/c nude mice (4–6 week-old) were subcutaneously inoculated with 5 × 10^7^cells of P-gp overexpressing LCC6MDR and allowed to develop tumor. Treatment began when a solid tumor of 150–200 mm^3^ was formed. Tumor-bearing mice were randomized into four groups with 7–8 mice in each group: (1) control group—no treatment, (2) PTX alone (12 mg/kg, I.V.), (3) PTX alone (24 mg/kg, I.V.) and (4) co-treatment: **FM04** (28 mg/kg, I.P.) was injected 1 hr before PTX (12 mg/kg, I.V.) administration. Tumor growth was measured by an electronic caliper on the injection day and the tumor volume was calculated using the following formula:

$$\mathrm{Estimated}\;\mathrm{tumor}\;\mathrm{volume}\;\left({\mathrm{mm}}^{3}\right)=\frac{l\times w^{2}}{2}$$

Statistical analysis on tumor volumes of different treatment groups was done by one-way ANOVA (*, *p* < 0.05; **, *p* < 0.01; ***, *p* < 0.001).

### 4.16. Pharmacokinetic (PK) Study of **FM04** and Its Effect on PK of PTX in Mice after Oral Co-administration

PTX drug solution was prepared in 5% Cremophor EL, 5% ethanol and 90% saline on the day of the experiment. PTX + **FM04** drug solution was prepared in 7% Cremophor EL, 8% ethanol and 85% saline on the day of the experiment. PTX drug solution or PTX + **FM04** mixture drug solution was administered to the Balb/c mice (female, 4–6 week-old) via oral gavage. Blood was collected via cardiac puncture at scheduled time points (10, 30, 60, 120, 240 and 420 min) under deep anesthesia. All animals were terminated at the end of the experiment. Plasma concentrations of **FM04** and PTX were detected using UPLC-MS/MS. Plasma concentration–time profiles were analyzed by non-compartmental analysis (NCA). The area under the plasma concentration–time curve by trapezoid rules (AUC) (from 0 to infinity), the terminal half-life (t_1/2β_), initial half-life (t_1/2α_), mean residence time (MRT), maximum observed concentration (C_max_) and time to maximum concentration (T_max_) were calculated by PK Solutions 2.0 (Summit Research Service, Ashland, Wilmington, DE, USA).

### 4.17. UPLC-MSMS Quantification of **FM04** and Paclitaxel (PTX)

[^13^C_6_]-paclitaxel (CAS: 379688-61-6) was the internal standard (IS) and purchased from Alsachim. Acetonitrile (ACN) and methanol used were HPLC grade and water was purified by the Milli-Q system. The deuterated benzyl form of **FM04** (**D7-FM04**) was synthesized in-house and used as the internal standard (IS). The purity of all compounds used in this project was > 98%. A stock solution of **FM04** was weighed and prepared in methanol and Milli-Q water (50:50, *v*/*v*) to a stock concentration of 10 µg/mL. Serial dilution of **FM04** stock solution in 50% methanol was done to obtain standard curve and quality control samples.

PTX and its internal standard (IS) separation were performed on Agilent 1290 series UPLC coupled with Agilent 6460 Triple quadrupole. The calibration curve of PTX was constructed by quantifying the known amount of PTX that spiked to blank mice plasma following identical sample preparation. The mass spectrometer was configured in ESI + ionization mode monitoring MRM ion transition of *m*/*z* 854 > 286 (PTX) and *m*/*z* 882 > 314 (IS). Source parameters were as follows, gas temp (350 °C), gas flow (8 L/min), nebulizer (35 psi) and capillary (4000 V). UPLC chromatographic separation was performed on Waters Acquity UPLC BEH C18 (1.7 µM, 2.1 × 50 mm) with a 5 min gradient elution of 30% acetonitrile with 0.1% formic acid to 76% ACN with 0.1% formic acid. Milli-Q with 0.1% formic acid was used as the aqueous phase. Each separation and re-equilibration were completed in 9 min.

Detection and quantification of **FM04** and its internal standard were done by using the UPLC-MS/MS system consist of an Acquity Waters UPLC interfaced with a triple quadrupole mass spectrometer (Micromass model Quattro Ultima) equipped with an electrospray ionization source in positive mode. The desolvation temperature, capillary voltage, cone gas and desolvation gas were 350 °C, 3 Kv, 150 L/h and 600 L/h, respectively. Multiple reaction monitoring (MRM) was set to monitor the transitions from 416 > 239 (**FM04**) and 423 > 239 (IS). Chromatography separation was done with an Acquity UPLC BEH C8 column (2.1 × 50 mm, 1.7 µM). The mobile phase consisted of methanol + 0.1% formic acid (solvent B) and Milli-Q water + 0.1% formic acid (solvent A). The flow rate was 0.4 mL/min. The initial condition was 90% solvent A and 10% solvent B. After 1 min elution by the initial condition, a linear gradient was performed with solvent B increasing from 10% to 100% for 10 min. Afterwards, the mobile phase was restored to the initial condition for re-equilibration. The total analysis time was 20 min per injection.

### 4.18. Effect of **FM04** on Transepithelial Transport of PTX Using Caco-2 Transwell Assay In Vitro

Caco-2 cells in between passage number 21 and 30 were used for the experiment, 1 × 10^5^ cells/cm^2^ Caco-2 cells were seeded in Costar 6-well transwell plate (Corning Inc). The insert membrane pore size was 0.4 μm with a growth area of 4.67 cm^2^. The culture medium was changed three times a week. Transport assays were performed 14 to 19 days post-seeding. The integrity of cell monolayers was evaluated on the day of experiments by measuring the trans-epithelial electrical resistance (TEER) of the cell monolayers. Only cell monolayers with TEER values above 500 Ω·cm^2^ were used for the transport experiment. Drug transport was evaluated at 37 °C by incubating the cells in media containing PTX (10 μM) with or without **FM04** (10 μM) at the donor chamber and samples collected at the receiver chamber. For apical to basolateral transport (A to B), treatment was added at the apical side and samples of the medium were collected at the basal side and vice versa for basal to apical transport (B to A). Samples of the medium were collected at 0 (pre-treatment), 20, 40, 60, 80, 100 and 120 min at the receiver compartment. The samples were stored at −20 °C until further analysis.

### 4.19. Effect of **FM04** on PTX Metabolism Using Human Liver Microsomes (HLM) Assay In Vitro

Drug solutions of PTX and **FM04** were prepared in methanol. PTX (10 μM) with or without **FM04** (10 or 20 μM) was firstly pre-incubated with 100 μL HLM reaction and the reaction was initiated by adding NADPH as mentioned previously. Protein precipitation by methanol was employed for sample clean-up. PTX and PTX metabolites were immediately analyzed by UPLC-MS/MS on the same day.

### 4.20. UPLC-MSMS Quantification of PTX and Its Metabolites

[^13^C_6_]-paclitaxel (CAS: 379688-61-6) was the internal standard (IS) and purchased from Alsachim. Fifteen microliters of 10 μg/mL internal standard (IS) was added to all samples and calibration standards for quantification. PTX and IS separation were performed on Agilent 1290 series UPLC coupled with Agilent 6460 Triple quadrupole. Serial dilution of PTX stock solution in ACN was used to construct the calibration curve. The mass spectrometer was configured in ESI+ ionization mode monitoring MRM ion transition of *m*/*z* 854 > 286 (PTX), *m*/*z* 870 > 302 (p3-OHP), *m*/*z* 870 > 525 (6α-OHP) and *m*/*z* 882 > 314 (internal standard). Source parameters were as follows, gas temp (350 °C), gas flow (8 L/min), nebulizer (35 psi) and capillary (4000 V). UPLC chromatographic separation was performed on Waters Acquity UPLC BEH C18 (1.7 µM, 2.1 × 50 mm) with a 9 min gradient elution of 40% acetonitrile (ACN) with 0.1% formic acid to 100% ACN with 0.1% formic acid. Milli-Q with 0.1% formic acid was used as the aqueous phase. Each separation and re-equilibration were completed in 16 min. PTX and both metabolites were semi-quantified base on the peak area ratio of the analyte/internal standard.

### 4.21. Efficacy of Orally Co-Administered PTX with **FM04** in Treating the Human Melanoma MDA435/LCC6 Xenograft Model In Vivo

All drug solutions were prepared freshly prior to oral administration. The formulation for PTX and **FM04** were described in the previous section.

LCC6 cells at 1 × 10^6^ were injected intraperitoneally (I.P.) to allow ascite development for cell adaptation in vivo. Aspirated ascite was then injected S. C. to nude mice for the development of solid tumors as stock. These solid tumors were excised and trimmed into 1 mm^3^ cubes for constructing subsequent S.C. xenografts. The study begun when these xenografts were confirmed to bear a tumor at 100–120 mm^3^. **FM04** (22 or 45 mg/kg) was co-administered orally with PTX (40, 60, 70 or 80 mg/kg). The treatment schedule was set to once a day for four times in two cycles (q1d × 4, 2 cycles). Tumor volume and body weight changes were monitored throughout the experiment. Animals with a body weight loss > 15% during treatment for more than 3 consecutive days were considered to be treatment-induced toxicity. All animals were sacrificed on day 30. Statistical analysis was performed by using one-way ANOVA (*, *p* < 0.05; **, *p* < 0.01; ***, *p* < 0.001).

## Figures and Tables

**Table 1:** EC_50_ (nM) of **FD18** metabolites in reversing P-gp mediated MDR.

		Compounds			
		FD18	14a (M1)	FM04 (M2)	FM327 (M3)
EC_50_ (nM) needed for reversing MDR in LCC6MDR cells	PTX	148 ± 18 ^a^	305 ± 35 ^a^	83 ± 7	>1000
	Vinblastine	173 ± 27 ^a^	ND	61 ± 13	ND
	Vincristine	179 ± 32 ^a^	ND	83 ± 11	ND
	DOX	131 ± 13 ^a^	ND	153 ± 39	ND
	Daunorubicin	95 ± 25 ^a^	ND	88 ± 52	ND
	Mitoxantrone	90 ± 20 ^a^	ND	64 ± 27	ND
Physicochemical properties	Molecular Weight	724	ND	415	ND
	CLogP	9.0	ND	4.9	ND
	tPSA (Å^2^)	92.8	ND	56.8	ND

Effective concentration (EC_50_) was a concentration of modulator at which the IC_50_ of an anticancer drug can be reduced by half in the LCC6MDR cells. ^a^ EC_50_ values of **FD18** or **14a** for reversing MDR in LCC6MDR cells had been published in 2012 [23]. They were included here for comparison. Molecular weight, CLogP and tPSA were compared for **FD18** and **FM04**. The CLogP and tPSA were analyzed using ChemDraw Ultra 12.0 software. The EC_50_ values were presented as mean ± standard error of the mean. N = 3–7 independent experiments. ND = not determined.

**Table 2:** Selectivity of **FM04** towards P-gp, BCRP and MRP1 transporters.

Compounds	IC_50_ of Anticancer Drugs (nM)					
	P-gp-Transfectant LCC6MDR		BCRP-Transfectant HEK293/R2		MRP1-Transfectant 2008/MRP1	
	Paclitaxel (nM)	RF	Topotecan (nM)	RF	DOX (nM)	RF
DMSO	129.6 ± 7.9	1.0	224.8 ± 32.0	1.0	616.0 ± 54.2	1
1 µM **FM04**	4.6 ± 0.5	28.2	131.7 ± 14.5	1.7	522.2 ± 27.6	1.2
1 µM Cyclosporine A	1.8 ± 0.5	72.0	/	/	/	/
1 µM Ko143	/	/	16.4 ± 1.2	13.7	/	/
1 µM **FD-4e**	/	/	/	/	44.5 ± 10.1	13.8

The selectivity of **FM04** was studied using P-gp-overexpressing cell line LCC6MDR, BCRP-overexpressing cell line HEK293/R2 and MRP1-overexpressing cell line 2008/MRP1. The IC_50_ of anticancer drugs in these cell lines was tested with or without 1 μM of the modulator. RF (relative fold): IC_50_ of anticancer drugs in a transfectant cell line without the modulator / IC_50_ of anticancer drugs in a transfectant cell line with the modulator. N = 2–8 independent experiments. The IC_50_ values were presented as mean ± standard error of the mean. Specific P-gp, BCRP and MRP1 inhibitors acted as a positive control in the study including cyclosporine A, Ko143 and **FD-4e** [26]. /: not determined.

## Data Availability

The data presented in this study are available on request from the corresponding author.

## Supplementary Materials

The following supporting information can be downloaded at: https://www.mdpi.com/article/10.3390/ijms232315299/s1.

## Abbreviations

P-gp	P-glycoprotein
MDR	multidrug resistance
PTX	paclitaxel
DOX	doxorubicin
AUC	area under the curve
I.P.	intraperitoneal
I.V.	Intravenous
BCRP	breast cancer resistance protein
MRP1	multidrug resistance associated protein 1
GI	gastrointestinal
