# Supplementary material for: Discovery of a Flavonoid FM04 as a Potent Inhibitor to Reverse P-Glycoprotein-Mediated Drug Resistance in Xenografts and Improve Oral Bioavailability of Paclitaxel
Source: Int J Mol Sci. 2022 Dec 4;23(23):15299. doi: 10.3390/ijms232315299 (PMC9739180; doi:10.3390/ijms232315299)
Supplement: Supplementary file 1 [file ijms-23-15299-s001.zip › ijms-1998948-supplementary.pdf]

Supplementary information

Discovery of a flavonoid FM04 as a potent inhibitor to reverse P-glycoprotein-mediated drug resistance in xenograft and improve oral bioavailability of paclitaxel

Jason W. Y. Kan[1#], Clare S. W. Yan[1#], Iris L. K. Wong[1], Xiaochun Su[1], Zhen Liu[1], Tak Hang Chan[1,2*] and Larry M. C. Chow[1*]

[1]Department of Applied Biology and Chemical Technology and State Key Laboratory of Chemical Biology and Drug Discovery, Hong Kong Polytechnic University, Hong Kong SAR, China.

[2]Department of Chemistry, McGill University, Montreal, Quebec, H3A 2K6 Canada.

#These authors contributed equally to this work.

*Co-corresponding authors: Tak Hang Chan and Larry M. C. Chow

For THC, Phone: 852-34008670; Fax: 852-23649932; E-mail: tak-hang.chan@polyu.edu.hk

For LMCC, Phone: 852-34008662; Fax: 852-23649932; E-mail: larrychow@polyu.edu.hk

Conflicts of Interest: The authors declare no potential conflicts of interest.

Table S1. IC_{50} of anticancer drugs in LCC6 and LCC6MDR and P-gp modulating activity of **FM04**.

Anticancer drugs	LCC6MDR + DMSO		LCC6MDR + 1 μM **FM04**		LCC6 + DMSO		LCC6 + 1 μM **FM04**	
	IC_{50} (nM)	RF	IC_{50} (nM)	RF	IC_{50} (nM)	RF	IC_{50} (nM)	RF
Paclitaxel	129.6 ± 7.9	1.0	3.8 ± 0.4	34.1	2.1 ± 0.5	61.7	3.0 ± 0.9	43.2
Vinblastine	9.2 ± 1.1	1.0	0.15 ± 0.02	61.3	0.38 ± 0.14	24.2	0.9 ± 0.3	10.7
Vincristine	27.9 ± 2.7	1.0	0.41 ± 0.03	68.0	0.71 ± 0.32	39.3	1.3 ± 0.3	21.5
DOX	1041.6 ± 104.0	1.0	41.3 ± 18.5	25.2	27.0 ± 4.6	38.6	/	/
Daunorubicin	1336.2 ± 227	1.0	210.8 ± 99.0	6.3	22.1 ± 2.2	60.5	/	/
Mitoxantrone	1052.4 ± 148	1.0	61.0 ± 2.3	17.3	37.4 ± 11.7	28.1	/	/

IC_{50} of anticancer drug with or without 1μM of **FM04** was determined in LCC6 and LCC6MDR. RF (relative fold): IC_{50} of an anticancer drug of LCC6MDR without **FM04**/IC_{50} of an anticancer of LCC6 with or without **FM04** or LCC6MDR with **FM04**. N = 2 - 8 independent experiment. The IC_{50} values were presented as mean ± standard error mean. /: not determined.

Table S2. Cytotoxicity of **FM04** and **FD18** towards LCC6, LCC6MDR and L929 cells.

Cell lines	IC_{50} (μM)	
	**FM04**	**FD18**
LCC6	10.3 ± 0.2	>75.0[a]
LCC6MDR	5.7 ± 1.5	>63.0[a]
L929	33.0 ± 0.0	85.0 ± 5.0[a]

The cytotoxicity of **FM04** and **FD18** had been tested using LCC6, LCC6MDR and L929. N = 2 -3 independent experiment. L929 is a mouse fibroblast cell line. The IC_{50} value was presented as mean ± standard error mean. [a] the data has been reported in 2012 [23]. They were included here for comparison.
